# Self-Assembled Particles Combining SARS-CoV-2 RBD Protein and RBD DNA Vaccine Induce Synergistic Enhancement of the Humoral Response in Mice

**DOI:** 10.3390/ijms23042188

**Published:** 2022-02-16

**Authors:** Mariya B. Borgoyakova, Larisa I. Karpenko, Andrey P. Rudometov, Ekaterina A. Volosnikova, Iuliia A. Merkuleva, Ekaterina V. Starostina, Alexey M. Zadorozhny, Anastasiya A. Isaeva, Valentina S. Nesmeyanova, Daniil V. Shanshin, Konstantin O. Baranov, Natalya V. Volkova, Boris N. Zaitsev, Lyubov A. Orlova, Anna V. Zaykovskaya, Oleg V. Pyankov, Elena D. Danilenko, Sergei I. Bazhan, Dmitry N. Shcherbakov, Alexander V. Taranin, Alexander A. Ilyichev

**Affiliations:** 1 State Research Center of Virology and Biotechnology “Vector”, 630559 Koltsovo, Novosibirsk Region, Russia; borgoyakova_mb@vector.nsc.ru (M.B.B.); rudometov_ap@vector.nsc.ru (A.P.R.); volosnikova_ea@vector.nsc.ru (E.A.V.); j.a.merkulyeva@gmail.com (I.A.M.); starostina_ev@vector.nsc.ru (E.V.S.); zadorozhnyy_am@vector.nsc.ru (A.M.Z.); isaevaanastasya93@gmail.com (A.A.I.); nesmeyanova_vs@vector.nsc.ru (V.S.N.); shanshin_dv@vector.nsc.ru (D.V.S.); volkova_nv@vector.nsc.ru (N.V.V.); zaitsev@vector.nsc.ru (B.N.Z.); orlova_la@vector.nsc.ru (L.A.O.); zaykovskaya_av@vector.nsc.ru (A.V.Z.); pyankov@vector.nsc.ru (O.V.P.); danilenko_ed@vector.nsc.ru (E.D.D.); bazhan@vector.nsc.ru (S.I.B.); dnshcherbakov@gmail.com (D.N.S.); ilyichev@vector.nsc.ru (A.A.I.); 2 Institute of Molecular and Cellular Biology, Siberian Branch of the Russian Academy of Science, 630090 Novosibirsk, Russia; baranov@mcb.nsc.ru (K.O.B.); taranin@mcb.nsc.ru (A.V.T.)

**Keywords:** SARS-CoV-2, self-assembled particles, RBD protein, DNA vaccine, humoral response, virus-neutralizing activity, cellular response

## Abstract

Despite the fact that a range of vaccines against COVID-19 have already been created and are used for mass vaccination, the development of effective, safe, technological, and affordable vaccines continues. We have designed a vaccine that combines the recombinant protein and DNA vaccine approaches in a self-assembled particle. The receptor-binding domain (RBD) of the spike protein of SARS-CoV-2 was conjugated to polyglucin:spermidine and mixed with DNA vaccine (pVAXrbd), which led to the formation of particles of combined coronavirus vaccine (CCV-RBD) that contain the DNA vaccine inside and RBD protein on the surface. CCV-RBD particles were characterized with gel filtration, electron microscopy, and biolayer interferometry. To investigate the immunogenicity of the combined vaccine and its components, mice were immunized with the DNA vaccine pVAXrbd or RBD protein as well as CCV-RBD particles. The highest antigen-specific IgG and neutralizing activity were induced by CCV-RBD, and the level of antibodies induced by DNA or RBD alone was significantly lower. The cellular immune response was detected only in the case of DNA or CCV-RBD vaccination. These results demonstrate that a combination of DNA vaccine and RBD protein in one construct synergistically increases the humoral response to RBD protein in mice.

## 1. Introduction

The ongoing COVID-19 pandemic, caused by severe acute respiratory syndrome coronavirus 2 (SARS-CoV-2), requires the development of new effective vaccines [1]. Some of the first to be licensed were messenger RNA (mRNA) vaccines, vector vaccines, and inactivated vaccines [2,3,4,5,6]. Vaccines based on other approaches are now emerging, including DNA vaccines and subunit vaccines [7].

The COVID-19 DNA vaccine, developed by Zydus Cadila (India), is the world’s first DNA vaccine to be approved for human vaccination [8,9]. Among the advantages of DNA vaccines, it should be noted that they, like vector and mRNA vaccines, efficiently induce T cell immunity, including T helper cells and cytotoxic T lymphocytes [10], while having a relatively good safety profile [11]. Moreover, it is cost-effective to produce DNA vaccines, they have the ability to be adapted rapidly to new targets, and they are stable at room temperature, all features that compare favorably with mRNA vaccines, which require storage at low temperatures [12,13,14]. A definite disadvantage of DNA vaccines is their low immunogenicity when injected as naked plasmid DNA [15,16]. A wide range of strategies have been tried to increase the immunogenicity of DNA vaccines, including packaging into liposomes; the use of “vaccine cocktails” containing the DNA vaccine as well as plasmids encoding adjuvant immunomodulatory proteins; and delivery by a gene gun, electroporation, or with a needle-free injector device [8,17,18,19]. These methods help to solve the problem of immunogenicity, but there are sometimes safety problems, technological difficulties, and an increase in the cost of the developed vaccine.

Subunit vaccines are another rapidly developing area in the development of COVID-19 prevention tools [20,21,22]. Currently, a number of subunit vaccines based on the spike (S) protein or its fragments are undergoing clinical trials [7,23,24]. Protein vaccines induce a weak cellular response, but they effectively stimulate the humoral B cell response. A recent review [25] suggests that protein vaccines may induce a longer humoral response against SARS-CoV-2 than nucleic acid and inactivated vaccines. An important factor affecting the efficiency of stimulation of the humoral response and neutralizing antibodies is the degree of multimerization of protein viral antigens. It has been shown that immunogens based on dimeric and multimeric derivatives of protein receptors (namely the receptor-binding domain (RBD)) induce a significantly higher titer of neutralizing antibodies [26,27].

A prime-boost strategy using a combination of DNA vaccines and recombinant proteins, which consists of priming the immune response with DNA vaccines and then boosting with a protein, allows an effective immune response to be achieved while overcoming the potential drawbacks of both approaches [28,29,30]. However, the idea of co-delivering DNA vaccines and proteins seems to be a simpler and more easily controlled method of immunization compared with the prime-boost regimen [31]. Several studies have shown that co-administration of DNA and protein in the same anatomical sites play a critical role in the development of protective immunity [32].

We suggest a novel concept of combining recombinant proteins and DNA vaccine in the form of artificial self-assembled particles. We have previously used this original approach to develop the candidate vaccine CombiHIVvac [33]. In this article, we constructed a combined vaccine against SARS-CoV-2. The RBD protein is conjugated to polyglucin:spermidine (PGS) and mixed with plasmid pVAXrbd, which leads to the formation of particles containing the DNA vaccine inside and RBD on the surface. We called this combined vaccine CCV-RBD. Here, we present the results of the immunogenicity of CCV-RBD in an animal model.

## 2. Results

### 2.1. Verification of RBD Gene Transcription in Transfected HEK293T Cells

The plasmid pVAXrbd construction was carried out as described in Section 4. To verify the expression of the *RBD* gene, HEK293T cells were transfected with pVAXrbd or pVAX (as a negative control).

**Figure 1:**
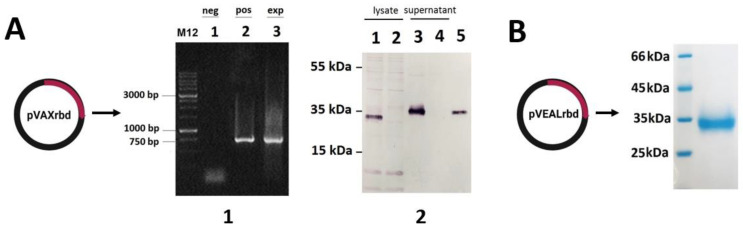
Verification of the RBD expression in transfected cells. (**A1**) HEK293T cells were transfected with pVAXrbd or pVAX (negative control). The target gene expression was confirmed with corresponding mRNA detection using RT-PCR. Electrophoretic analysis of RT-PCR products in 1% agarose gel: lanes 1 and 3 are products obtained from total RNA of HEK293T cells transfected with pVAX and pVAXrbd, respectively; lane 2 is the product obtained by PCR of plasmid pVAXrbd. (**A2**) Analysis of the RBD protein production by Western blot in HEK293T cells: lanes 1 and 2 are lysates of HEK293T transfected with pVAXrbd and pVAX, respectively; lanes 3 and 4 are culture medium from HEK293T cells transfected with pVAX and pVAXrbd, respectively; lane 5 is purified recombinant RBD. (**B**) SDS-PAGE analysis of purified recombinant RBD produced in CHO-K1 cells transfected with pVEALrbd.

Using the total RNA isolated from the transfected cells, we confirmed the expression of the *RBD* gene by RT-PCR. The presence of a specific 750 base pair (bp) product indicates the transcription of the transgene in plasmid pVAXrbd (Figure 1(A1)). As shown in Figure 1(A2), Western blot analysis confirmed the expression of the SARS-CoV-2 RBD protein at the expected molecular weight in the lysate and in the culture medium of the transfected cells (lanes 1 and 3). The lysate and culture medium of HEK293T transfected with plasmid pVAX (the negative control) did not show any other proteins specifically reactive with the mouse anti-SARS-CoV-2 antibody (Figure 1(A2), lanes 2 and 4).

### 2.2. Preparation and Characterization of the Particles

**Figure 2:**
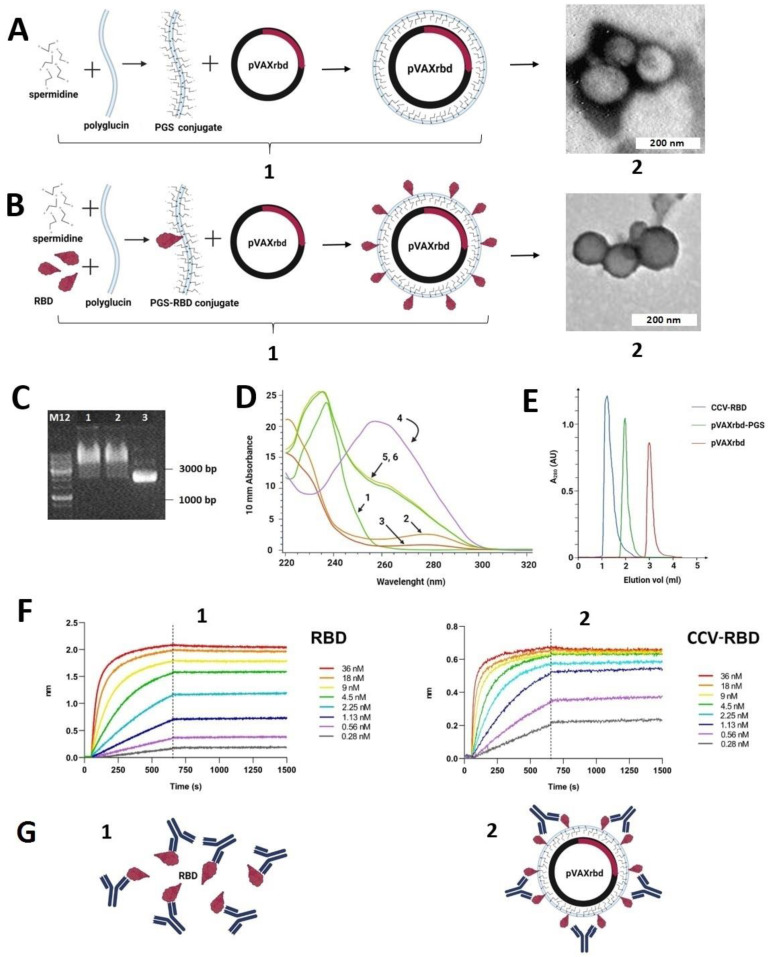
General scheme for DNA/protein complexation and characterization of their components. (**A1**) Schematic representation of the pVAXrbd-PGS particle (control) assembly. (**A2**) Electron micrograph of pVAXrbd-PGS particles. (**B1**) Schematic representation of the particle assembly of the combined CCV-RBD vaccine. (**B2**) Electron micrograph of CCV-RBD particles. (**C**) Confirmation of DNA encapsulation in the shell of PGS and PGS-RBD by electrophoresis in a 1% agarose gel: 1, CCV-RBD; 2, pVAXrbd-PGS; and 3, naked plasmid pVAXrbd. (**D**) UV spectra of the CCV-RBD and pVAXrbd-PGS structures and their components. 1, PGS; 2, RBD; 3, PGS-RBD; 4, pVAXrbd plasmid; 5 and 6, CCV-RBD and pVAXrbd-PGS particles, respectively. (**E**) Gel filtration on a Sepharose CL-6B column (chromatographic profile). Blue line, CCV-RBD; green line, pVAXrbd-PGS; red line, pVAXrbd plasmid. (**F1**) Octet binding of the RBD to iB14, SARS-CoV-2 nAb. (**F2**) Octet binding of the CCV-RBD to iB14, SARS-CoV-2 nAb. (**G1**) Schematic representation of RBD protein and antibody interaction. (**G2**) Schematic representation of CCV-RBD particle and antibody interaction.

We generated two types of particles: (1) CCV-RBD, particles that contain plasmid DNA encapsulated in the RBD protein conjugated with PGS (PGS-RBD) (Figure 2(B1)), and (2) pVAXrbd-PGS (control), particles that contain plasmid DNA encapsulated in PGS without protein (Figure 2(A1)).

Before obtaining the particles, we characterized their components apart. The RBD protein was analyzed by sodium dodecyl sulfate–polyacrylamide electrophoresis (SDS-PAGE); it was >98% pure (Figure 1B) [34]. The preparation of the PGS-RBD conjugate is described in Section 4. The presence of protein in PGS-RBD was also demonstrated by using ultraviolet (UV) spectroscopy (Figure 2B): arrows 2 and 3 indicate the spectra of RBD and PGS-RBD, respectively, which have an absorption peak at 280 nm, characteristic of proteins.

Particles were obtained via self-assembly by mixing PGS-RBD (or PGS) with plasmid pVAXrbd as described in Section 4. The formation of self-assembled particles was assessed by an electrophoretic mobility shift in the agarose gel: The encapsulated plasmid lost its mobility in the electric field when coated with either conjugate (PGS or PGS-RBD) (Figure 2C). Maintenance of the plasmid DNA structure alone and in the CCV-RBD and pVAXrbd-PGS complexes was confirmed by UV spectroscopy (Figure 2D, arrows 4, 5, and 6, respectively): the spectra of these preparations have a characteristic DNA peak at 260 nm.

Particle formation was also confirmed by gel filtration on Sepharose CL-6B. As shown in Figure 2E, the peak corresponding to the CCV-RBD fraction emerges in the free volume, ahead of pVAXrbd-PGS and plasmid DNA. We also examined the resulting particles by using electron microscopy (Figure 2(A2,B2)).

We evaluated the binding efficiency of anti-RBD neutralizing Ab (nAb) iB14 to RBD [35] by using the Octet RED96 instrument (ForteBio, Pall Life Sciences) to confirm that the RBD protein present on the particle surface retained its antigenic properties and was available for interaction with antibodies (Figure 2F). The binding of nAb iB14 to an epitope which is partially overlapped with the RBD-ACE2 interface [35] quite effectively recognized RBD in CCV-RBD, with a dissociation constant of 6.6 × 10^−11^ compared with 1.47 × 10^−10^ for the RBD protein alone.

### 2.3. Humoral Immune Response

**Figure 3:**
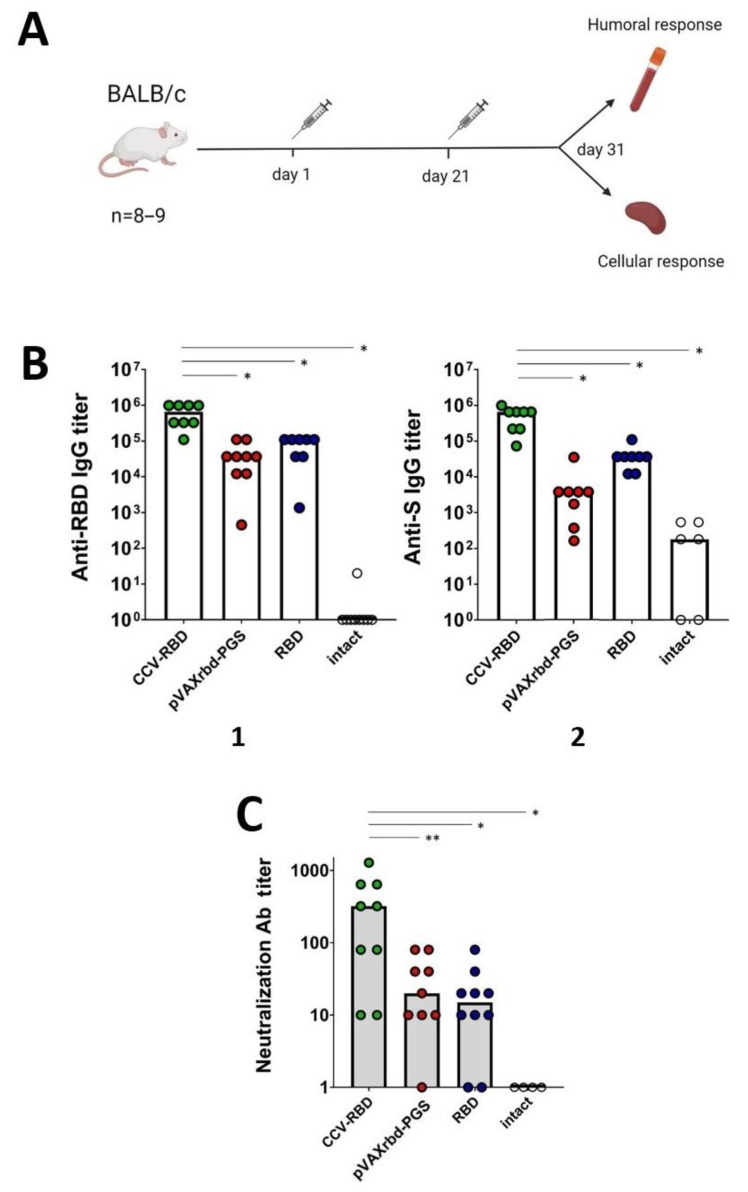
Humoral immune response in BALB/c mice. (**A**) Mice were immunized intramuscularly with CCV-RBD, pVAXrbd-PGS, or RBD protein twice on days 0 and 21 and serum samples were collected 10 days after the second immunization (day 31). (**B**) Titers of specific IgG antibodies to SARS-CoV-2 RBD (**1**) and SARS-CoV-2 S (**2**) were determined by ELISA. (**C**) The virus-neutralizing activity of sera from mice immunized with CCV-RBD, pVAXrbd-PGS, and RBD protein was determined by using the SARS-CoV-2 nCoV/Victoria/1/2020 strain (100 TCID_50_). In panels (**B**,**C**), data are presented as median reciprocals of titers. Significance was calculated using nonparametric Mann-Whitney method (* *p* < 0.01, ** *p* < 0. 05).

To assess the immunogenicity of the created vaccine constructs, BALB/c mice were immunized twice, on days 0 and 21. At the endpoint (day 31, 10 days after the final immunization), serum samples were collected and tested by enzyme-linked immunosorbent assay (ELISA) for the presence of antibodies specifically recognizing RBD and S proteins, as well as the ability of sera to neutralize live virus. Sera from mice immunized with pVAXrbd-PGS and the RBD protein, as well as sera from intact mice, were used as controls (Figure 3A).

According to the results of the RBD-specific ELISA at the endpoint of the experiment, there was a >32-fold increase in the median titer of specific antibodies in animals immunized with the combined DNA/protein vaccine CCV-RBD compared with the group that was injected with pVAXrbd-PGS, and there was an 8-fold increase compared with the group treated with the RBD protein without adjuvants (Figure 3(B1), *p* < 0.01). The S-specific ELISA—to detect the S protein—showed a 75-fold increase in the CCV-RBD-immunized group compared with the group that was injected with pVAXrbd-PGS, and a 13-fold increase compared with the RBD-immunized group (Figure 3(B2), *p* < 0.01).

The neutralizing properties of sera were analyzed by using the SARS-CoV-2 nCoV/Victoria/1/2020 strain in cell culture in vitro. The results of the analysis correlate with the results of ELISA: the sera of animals immunized with the CCV-RBD showed a higher activity in inhibiting the cytopathic effect of the virus (Figure 3C, *p* < 0.05).

Thus, immunization with the combined DNA/protein vaccine CCV-RBD induced a greater humoral immunity (IgG and neutralizing antibodies) than immunization with either DNA or the RBD protein alone. 

### 2.4. Cellular Immune Response

To assess the ability of the created vaccine constructs to induce cellular immunity in mice, spleens were taken at the endpoint of the experiment, homogenized, and examined by using the ELISpot method. Splenocytes of mice immunized with the PGS-encapsulated plasmid or RBD protein, as well as intact mice, were used as controls. The response was assessed by the ability of splenocytes to release interferon (IFN)-γ after specific stimulation, which was carried out by a pool of peptides that make up the RBD protein (Table 1).

**Figure 4:**
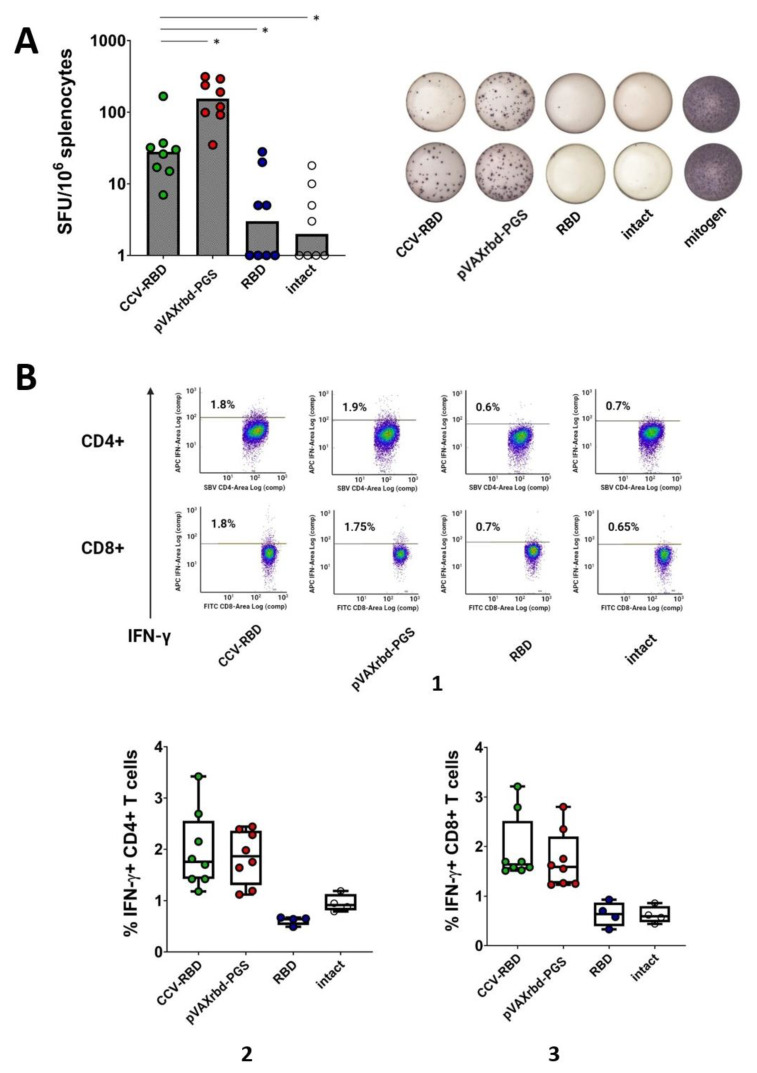
Cellular immune response in BALB/c mice. (**A**) The number of splenocytes releasing IFN-γ in response to specific stimulation with peptides from the RBD protein was counted in an ELISpot. (**B**) The percentage of SARS-CoV-2-specific IFN-γ-producing CD4+ (**2**) and CD8+ (**3**) T cells was analyzed by ISC and flow cytometry (**1**). Statistical analysis was performed by using GraphPad Prism 6.0 software. The significance of differences between samples was determined by using the nonparametric Mann–Whitney method (* *p* < 0.01).

Using ELISpot, the group immunized with the PGS-coated DNA vaccine showed the greatest cellular immunity (Figure 4A). In contrast to the humoral response, we did not observe an increase in cellular immunity in the CCV-RBD group. However, it should be noted that immunization with the RBD protein alone did not induce a significant cellular immune response.

To gain a more complete understanding of changes in T cell subpopulations, we assessed the percentage of IFN-γ-producing lymphocytes among all CD4+ and CD8+ T cells by intracellular cytokine staining (ICS) using flow cytometry. As shown in Figure 4B, after stimulation with peptides, CD4+ and CD8+ T lymphocytes responded with greater IFN-γ release in the groups immunized with the combined and DNA vaccines. The induction of cellular immunity, characterized by the production of IFN-γ, did not occur for the group immunized with the RBD protein or the intact group.

## 3. Discussion

The worldwide spread of SARS-CoV-2 has necessitated the rapid development of vaccines. The global community vaccination campaign has provided a unique opportunity to compare the different strategies and platforms of the developed vaccines. mRNA and vector vaccines remain the leaders at the moment, but new vaccines are entering the market—for example, based on the DNA platform [9]. There is also work in which it is predicted that the next generation of prophylactic COVID-19 vaccines will be protein vaccines [25]. In this work, to create a candidate vaccine against SARS-CoV-2, we have combined two platforms—a DNA vaccine and a recombinant protein. The developed combined vaccine against COVID-19, CCV-RBD, is an artificial particle containing the pVAXrbd DNA vaccine as a core, which encodes the receptor-binding domain of the S protein (Figure 1A), and on the surface the RBD protein is conjugated with the polymer PGS, as a particle envelope (Figure 2(B1)). RBD was chosen as the antigen because this region of the S protein is the dominant target for a neutralizing response in COVID-19 infection [36,37,38,39]. In addition, there are concerns that the full-length S protein may lead to increased viral infection or pulmonary toxicity [40,41].

To create virus-like particles, we encapsulated DNA vaccines in a PGS and RBD protein conjugate (Figure 2B). We have previously used the PGS conjugate to deliver candidate DNA vaccines against HIV-1 and Ebola [33,42,43]. We used the PGS-TBI protein conjugate to create a vaccine against HIV-1, namely CombiHIVvac, which has successfully passed the first phase of clinical trials [44]. These studies have demonstrated the safety of the PGS-TBI protein conjugate complex and a DNA vaccine. It is important to note that the PGS components are biodegradable and safe for humans; their low cost, safety, and the possibility of lyophilization with long-term storage provide additional technological advantages in the production and transportation of the vaccine preparation [45]. 

The spermidine molecules in the PGS and PGS-RBD conjugates provide a positive charge to these polymers. When negatively charged plasmid DNA is added to such conjugates, self-assembly of the complexes occurs: see Figure 2(A1), showing DNA in the PGS envelope, and Figure 2(B1), PGS-RBD-coated DNA. Particle formation was confirmed by changing the mobility of DNA in an agarose gel (Figure 2C) as well as by gel filtration (Figure 2E). Exposure of the RBD protein on the surface of particles was shown when studying the kinetics of binding to SARS-CoV-2 nAb iB14 [35] using an Octet RED96 device (Figure 2F). The lower Kd for CCV-RBD with iB14 nAb (6.6 × 10^−11^) compared with RBD alone (1.47 × 10^−10^) indicates a higher degree of binding of the protein in the particle to the nAb, which is apparently due to its multimeric representation on the surface of the particles (Figure 2F). 

The obtained CCV particles were 100–500 nm, a size comparable to viral particles (Figure 2(A2,B2)). This was confirmed by electron microscopy and gel filtration. A number of researchers have shown that nanoparticles of this size are optimal for creating vaccines, because they accumulate in B cell follicles and cause a strong immune response [46,47].

The study of the immunogenicity of CCV-RBD showed that the combined DNA/protein vaccine is able to induce a strong humoral response in immunized animals. The antibody titer in ELISA for the S-protein was 1:500,000 and the titer for RBD was 1:600,000. It is worth noting a synergistic increase (by one order of magnitude) of the humoral response to the administration of the combined vaccine compared with the groups that received the individual components: PGS-coated pVAXrbd and RBD protein (Figure 3B). The synergistic effect was even more pronounced in the study of the neutralizing activity of sera, which is the most important criterion for the effectiveness of the vaccine. The sera of all groups, except for intact mice, demonstrated the ability to neutralize the SARS-CoV-2 nCoV/Victoria/1/2020 strain in an in vitro cell culture in a virus-neutralization reaction (Figure 3C). However, in the CCV-RBD group, the mean neutralizing titer was 1:500, which is significantly higher than the titers shown in the pVAXrbd-PGS and RBD groups (1:40 and 1:30). The observed synergistic effect on the humoral response may indicate that antigen-presenting cells (APC) efficiently capture virus-like particles of CCV, as well as the stimulation of the T helper cell response due to the DNA component. The presence of multiple RBD proteins on the particle surface may also play a role in enhancing the humoral immune response to CCV-RBD. It should be noted that similar results were obtained earlier when we studied the immunogenicity of the candidate vaccine CombiHIVvac. Combining the conjugate PGS with TBI protein and the DNA vaccine, encoding T cell HIV-1 immunogen, in one construct exerted a synergistic effect on the induction of a B cell-mediated response [48].

When assessing the cellular response based on the number of splenocytes producing IFN-γ, determined by using the ELISpot method, the highest level of specific cellular immunity was found in the group immunized only with the RBD DNA vaccine coated with PGS (Figure 4A). However, analysis of CD4+ and CD8+ T cell subpopulations showed an approximately equal percentage of specific T lymphocytes in the groups immunized with DNA and combined vaccines (Figure 4(B2,B3)). Mice immunized with the CCV-RBD vaccine candidate showed a lower cellular response, while the response of the group immunized with protein alone was close to the response of non-immunized animals (Figure 4).

Taken together, the candidate CCV-RBD combined vaccine has several unique properties. It combines the RBD protein and the DNA vaccine in one construct and is a virus-like particle (based on using the conjugate of RBD protein with PGS). This design exhibits the advantages of a DNA vaccine (the ability to induce a cellular response) and a recombinant protein (capable of inducing a humoral response). Immunization with the combined construct results in a synergistic effect on the humoral immune response. The level of antibodies, including neutralizing ones, turns out to be several times higher than when immunized with the same doses of DNA or protein. This effect appears to be related to the particle size, the multimeric presentation of the RBD protein on the particle surface, and the T helper support provided by the DNA vaccine.

In addition, particles that present multiple copies of the antigen are more immunogenic than monomeric proteins due to the clustering of B cell receptors, the increased avidity of multimeric proteins, and the augmented retention of nanoparticles above 20 nm in the lymph nodes. Researchers have used several strategies to generate VLPs carrying immunogenic domains attached to natural or engineered protein scaffolds, such as the capsid proteins of viruses (HBcAg, HPV, JCV, bacteriophages, cowpea chlorotic mottle virus), proteins such as ferritin, encapsulin, lumazine synthase and others [47]. However, in this case, antibodies are also generated against the scaffold, which may reduce the effectiveness of subsequent vaccination with the same type of vaccine. The advantage of our vaccine platform in comparison with other VLP strategies is that we used dextran as the carrier, which is not immunogenic, and the immune response is generated only against the target antigen. The inclusion of a plasmid DNA vaccine into the core of the particles provides an additional adjuvant effect on antibody synthesis.

CCV-RBD is a promising vaccine candidate for use in the prevention of COVID-19, and the platform used to create combined vaccines will be useful for the design of various preventive or therapeutic drugs with the ability to induce humoral and cellular responses for protection.

## 4. Materials and Methods

### 4.1. Viruses and Cell Cultures

The HEK293T and Vero E6 cell lines were obtained from the collection of cell cultures of the SRC VB “Vector”, Koltsovo, Russia. The Vero cells were maintained in Dulbecco’s Modified Eagle Medium (DMEM) supplemented with 10% fetal bovine serum (FBS), 100 IU/mL penicillin, and 100 μg/mL streptomycin at 37 °C in the presence of 5% CO_2_. HEK293T cells were maintained in DMEM supplemented with GlutaMAX (Thermo Fisher Scientific, Waltham, MA, USA), 10% FBS, and 50 μg/mL gentamicin at 37 °C in the presence of 5% CO_2_.

The SARS-CoV-2 nCoV/Victoria/1/2020 strain was used in this work (State Collection of Causative Agents of Viral Infections and Rickettsioses, SRC VB “Vector,” Rospotrebnadzor, Koltsovo, Russian Federation). The virus was accumulated in a Vero E6 cell culture with a titer of 6.5 lg TCID_50_/mL in a Biosafety Level 3 (BSL-3) laboratory.

### 4.2. DNA Vaccine Construction and Protein Production

To construct plasmids, the *SARS-CoV-2 S* gene sequence was used (GenBank MN908947), specifically a fragment corresponding to the RBD protein (320V–542N). Optimization of the codon composition of the sequence was carried out by using the GeneOptimizer program (https://www.thermofisher.com/ru/en/home/life-science/cloning/gene-synthesis/geneart-gene-synthesis/geneoptimizer.html, accessed on 14 February 2022) for the expression of plasmids in CHO cells. The final nucleotide sequence was synthesized and cloned into the vector plasmid pVEAL2 as described earlier [34]. A fragment encoding the signal sequence of tissue plasminogen activator (Tpa) (MDAMKRGLCCVLLLCGAVFVSA) was added to the N-terminal region of the *RBD* gene using appropriate primers, and a fragment encoding a 6×His was added to the C-terminal region. The resulting integrative plasmid pVEALrbd was used to transfect CHO cells to generate an RBD protein producer. 

To obtain a DNA vaccine, the gene sequence encoding the RBD protein was synthesized and cloned into the pVAX vector under the immediate-early promoter of human cytomegalovirus (CMV). A fragment encoding a sequence that is a hybrid of fibroin and luciferase signal sequences (MMRTLILAVLLVYFCATVHC) was added to the N-terminus of the *RBD* gene. As a result, the pVAXrbd DNA vaccine was obtained.

### 4.3. In Vitro Investigation of Transgene Expression

Plasmid isolation and purification were carried out as described earlier [43]. Transfection of HEK293T cells with plasmid pVAXrbd was performed by using Lipofectamine 3000 (Invitrogen, Waltham, MA, USA) according to the manufacturer’s instructions. Cells transfected with plasmid pVAX were used as a negative control. The cells were seeded in 24-well plates at 1 × 10^7^ cells per well and cultured in DMEM supplemented with 10% FBS. On the day of transfection, the medium was replaced with a maintenance medium containing 2% fetal calf serum. Two micrograms of plasmid DNA with Lipofectamine 3000 was added to the plate well, and the plate was incubated at 37 °C in 5% CO_2_ atmosphere. 

Forty-eight h after transfection, HEK293T cells were harvested and total cell RNA was isolated by using an RNA isolation kit (OOO Biolabmix, Novosibirsk, Russia) and reverse transcription and PCR were carried out in a one-tube reaction by using an RT-PCR kit (OOO Biolabmix, Russia) with *RBD* gene specific primers SE-F (5′-taatacgactcactataggctagcct-3′) and SE-R (5′-aaaaaagcggccgctcattagttgaagttcacgcatttgttcttc-3′). Using a Verity 96-Well Thermal Cycler (Applied Biosystems, Waltham, MA, USA), the samples were subjected to the following thermal cycling: 30 min at 45 °C; 5 min at 95 °C; 30 cycles of 15 s at 95 °C, 20 s at 58 °C, and 2 min at 72 °C; and a final elongation for 5 min at 72 °C. The PCR products were analyzed on a 1% agarose gel.

Transfected HEK293T cells pellet and culture medium were used for Western blot analysis to investigate RBD protein production. Cells were lysed by using a Soniprep 150 Plus homogenizer (MSE, Nuaillé, France). Ten microliters of cell lysates and the culture medium in which the transfected cells were grown were loaded into the wells of 12% polyacrylamide gel, followed by electrophoresis and the transfer of proteins onto a nitrocellulose membrane (Hybond-C Extra, Amersham Bioscience, Buckinghamshire, UK) on a semi-dry transfer system (Bioclon, Moskow, Russia). The membrane was incubated with bovine serum albumin for 1 h at 37 °C to block nonspecific protein binding. After washing, the membrane was incubated with anti-SARS-CoV-2 hyperimmune mouse serum diluted 1:5000. After several washes, the membrane was incubated with rabbit anti-mouse IgG conjugated to alkaline phosphatase (Sigma, Burlington, MA, USA) for 1 h at room temperature. The membrane was then incubated with 5-bromo-4-chloro-3′-indolyl phosphate/nitro blue tetrazolium (BCIP/NBT) to visualize the protein bands.

### 4.4. Purification of the Recombinant RBD Protein

To generate an RBD protein-producing cell line, CHO-K1 cells were transfected with plasmid pVEALrbd by using Lipofectamine 3000 (Thermo Fisher Scientific) as described earlier [34]. To integrate the vector expression cassette into the cell genome, plasmid pCMV (CAT) T7-SB100 encoding SB100 transposase was added together with the target plasmid. After 3 days, the selective antibiotic puromycin (InvivoGen, San Diego, CA, USA) was added to the culture medium at a final concentration of 10 μg/mL; the pVEAL2 vector contains the puromycin resistance gene. The selection of resistant clones lasted for 3 days. The clones that showed the highest productivity were cultivated on roller installations, and the culture medium was harvested.

Recombinant RBD was isolated from the culture medium of CHO-K1 cells. It was centrifuged, filtered (0.22 μM), and subjected to two-stage chromatographic purification. The first stage included metal-chelate chromatography on a Ni-NTA column (Qiagen, Hilden, Düsseldorf, Germany) according to the manufacturer’s instructions. The next purification was carried out by ion-exchange chromatography on columns connected in series with cation-exchange (SP-Sepharose) and anion-exchange (Q-Sepharose) sorbents in 20 mM Tris-HCl (pH 8.2). The resulting protein was dialyzed against phosphate-buffered saline (PBS) and subjected to sterilizing filtration through 0.22 µm filters. The protein concentration was determined by using the Lowry method. The RBD protein preparation was analyzed by denaturing electrophoresis in 15% PAGE.

### 4.5. Synthesis of PGS and PGS-RBD Conjugates

We obtained two conjugates based on PGS, namely with and without protein (RBD). These conjugates were synthesized according to the same scheme with some differences. For the first stage, dextran was activated as follows: 1 mol of dextran 40,000 (MP Biomedicals, Irvine, CA, USA) was treated with 40 mol of sodium periodate for 60 min, after which time the remaining oxidizing agent was removed from the activated dextran by gel filtration on a column with Sephadex G-25 equilibrated with 50 mM carbonate buffer (pH 8.6). Next, RBD protein was added to the activated dextran solution—1 mol of dextran and 1 mol of protein—and the mixture was incubated for 2 h. Sodium borohydride was then added to the mixture at the ratio of 80 mol of borohydride per 1 mol of dextran. A conjugate without protein was obtained by excluding it from the mixture. After a 2 h incubation, the resulting conjugates were purified from unreacted components by gel filtration on Sephadex G-25 equilibrated with PBS. The conjugate preparation was sterile filtered through 0.22 µm filters.

### 4.6. DNA Packaging into PGS and PGS-RBD Conjugates

To form the pVAXrbd-PGS complexes, plasmid pVAXrbd was mixed with the PGS conjugate in a mass ratio of 1:10 [49]. To form particles of the combined CCV-RBD vaccine, the plasmid was mixed with the PGS-RBD conjugate at the ratio of 100 μg of DNA for every 100 μg of protein, incubated for 5 min, and then an excess of PGS was added so that the positive charge from the spermidine molecules was sufficient for electrostatic interaction with the negatively charged nucleic acid conjugate and to ensure complete wrapping around the plasmid (incubated for 1 h at 2–8 °C). The efficiency of the formation of the pVAXrbd-PGS and CCV-RBD complexes was assessed by the change in the electrophoretic mobility of DNA in a 1% agarose gel.

To evaluate the size and shape of the obtained particles, their suspensions were applied to copper grids for electron microscopy, covered with a carbon-stabilized formvar film. The preparations were stained with a 2% aqueous solution of phosphotungstic acid and examined using a JEM-1400 electron microscope (JEOL, Akishima, Tokyo, Japan). A Veleta digital camera (SIS, Schwentinental, Germany) was used to acquire images, and the iTEM software package (SIS, Schwentinental, Germany) was used to analyze and process the images.

Gel filtration of CCV-RBD, pVAXrbd-PGS, and pVAXrbd was carried out on a 10 mL Sepharose CL-6B column in PBS (pH 7.4). Samples of the studied preparations were applied in equimolar amounts to the nucleotide material.

UV absorption spectra of CCV-RBD, pVAXrbd-PGS, and their individual components (PGS, RBD, PGS-RBD, and plasmid pVAXrbd) were studied by using a Nanodrop One spectrophotometer in the wavelength range of 220–300 nm (Thermo Fisher Scientific).

### 4.7. Biolayer Interferometry

The kinetics of vaccine antigen (CCV-RBD), the recombinant fragment of the SARS-CoV-2 spike protein (RBD) versus the SARS-CoV-2 RBD-specific antibody iB14, was measured using an Octet RED96 instrument (ForteBio, Pall Life Sciences, NY, USA). All assays were performed with agitation set to 1000 rpm in ForteBio 1× kinetic buffer (PBS, 0.05% Tween, 0.05% NaN3). The final volume for all the solutions was 200 mkl per well. Assays were performed at 30°C in solid black 96-well plates (Greiner Bio-One, Kremsmünster, Austria). The ligands’ (CCV-RBD and RBD) 1× kinetic buffer was loaded onto the surface of Ni-NTA Biosensors (NTA) for 300 s. A 60 s biosensor baseline step was applied before the analysis of the association of the antigen on the biosensor to the antibody in solution for 600 s. A twofold concentration gradient of iB14 antibody, starting at 144 nM for RBD and CCV-RBD, was used in a titration series of eight. The dissociation of the interaction was followed for 900 s. The correction of baseline drift was performed by subtracting the mean value of shifts recorded for a sensor loaded with antigen, but not incubated with antibody, and for a sensor without antigen but incubated with antibody. Octet data were processed by ForteBio’s data acquisition software v.11.1.1.19. Experimental data were fitted with the binding equations describing a 2:1 interaction (Global fitting, Bivalent Analyte) to achieve optimal fitting.

### 4.8. Mouse Immunization

Work with animals was carried out in accordance with the “Guidelines for the Care and Use of Laboratory Animals”. The protocols were approved by the Institutional Animal Care and Use Committee (IACUC) at the State Research Center of Virology and Biotechnology “Vector” (permission number: SRC VB “Vector”, accessed on 9 October 2020).

To assess the immunogenicity of the created constructs, female BALB/c mice weighing 16–18 g were used. The mice were divided into four groups of 8–9 animals each and immunized as follows: CCV-RBD group, a combined vaccine containing 100 μg of DNA and 100 μg of protein; pVAXrbd-PGS group, 100 μg of plasmid pVAXrbd encapsulated in a PGS envelope; RBD group, 100 μg of recombinant RBD protein; intact group, no immunization. Animals of the first three groups received preparations in a volume of 200 μL injected intramuscularly in the upper part of the hind limbs (100 μL per limb).

Mice were immunized twice with an interval of 3 weeks between immunizations. Ten days after the second immunization, blood was taken from the animals to analyze the humoral immune response, and the spleen was collected to analyze the cellular response.

Serum was separated from cellular elements by centrifugation (9000× *g*, 15 min), heated for 30 min at 56 °C, and examined for the presence of antibodies that specifically bind to the S and RBD proteins in ELISA. The samples were also analyzed for their neutralizing activity. 

Spleens were sequentially minced on nylon filters for cells with pore diameters of 70 and 40 μm (BD Falcon, Franklin Lakes, NJ, USA). After lysis of erythrocytes with lysis buffer (Sigma, Burlington, MA, USA), splenocytes were washed twice in complete RPMI medium and placed in 1 mL of RPMI medium with 2 mM L-glutamine, 50 μg/mL gentamicin, and 10% FBS (Thermo Fisher Scientific, USA). Cells were counted by using a TC20 automatic cell counter (Bio-Rad, Hercules, CA, USA). Among the splenocytes, the number of IFN-γ-producing cells was determined by using the ELISpot method.

### 4.9. ELISA

Endpoint titers of IgG in sera from immunized mice were measured by ELISA. Recombinant eukaryotic RBD and S proteins were used as antigens, adsorbed to 96-well plates (Corning, NY, USA) at a concentration of 1 μg/mL in 2 M urea overnight at 4 °C. The plates were washed three times and incubated for 1.5 h at room temperature with 1% casein in PBS with 0.05% Tween 20 to block nonspecific protein binding. Then, the plates were incubated with threefold serial dilutions of mouse sera for 1 h at room temperature, and after washing with PBS with Tween 20, rabbit anti-mouse IgG conjugated to horseradish peroxidase (Sigma), diluted 1:3000, was added and incubated for 1 h at room temperature. After the final wash, the plates were developed using by using the substrate TMB (Amresco, Radnor, PA, USA). The reaction was stopped with 1 N HCl and analyzed at 450 nm on a Varioskan LUX multimode microplate reader (Thermo Fisher Scientific, Waltham, MA, USA).

### 4.10. Virus-Neutralizing Assay

The neutralizing properties of antibodies of blood serum were determined by inhibition of the cytopathic effect (CPE) of the virus in cell culture in vitro, as described previously [43]. Briefly, the SARS-CoV-2 nCoV/Victoria/1/2020 strain (100 TCID_50_), incubated with serial dilutions of the studied sera, was plated on a 100% confluent monolayer of Vero E6 cells. The plates were incubated for 4 days at 37 °C in the presence of 5% CO_2_, and then the cells were stained with 0.2% gentian violet solution. The results were recorded visually. The neutralizing activity of the sera of immunized animals was assessed by the titer (dilution) of sera at which the protection of cells from the CPE of the virus was recorded in 50% of the wells.

### 4.11. IFN-γ ELISpot

Analysis of the T cell immune response was performed by using the Mouse IFN-gamma ELISpot Kit (R&D Systems, Minneapolis, MN, USA) according to the manufacturer’s instructions. Splenocytes were plated at 5 × 10^5^ cells/well, and RPMI medium with 10% FBS (the negative control) or a mixture of peptides (each at a concentration of 20 μg/mL) or Concanavalin A (the positive control) was added to them. The cells were incubated for 20 h at 37 °C in the presence of 5% CO_2_. Subsequently, the plates were washed and incubated with the primary antibody against IFN-γ. The plates were washed again and then incubated with a secondary antibody conjugated to alkaline phosphatase. Finally, the plates were washed again and then incubated with BCIP/NBT. The number of IFN-γ-producing cells was counted by using an ELISpot reader (Carl Zeiss, Oberkochen, Germany). The number of spot-forming units (SFU) per million cells was calculated by subtracting the average value from the negative control wells.

Splenocytes isolated from immunized BALB/c mice were stimulated with a pool of peptides from the SARS-CoV-2 S protein sequence, restricted by major histocompatibility complex (MHC) class I (H2-Dd, H-2-Kd, and H-2-Ld) and MHC class II (H2-IAd and H2 -IEd) molecules of BALB/c mice (Table 1). The peptides were selected by using the IEDB Analysis Resource instruments and synthesized by AtaGenix Laboratories (Wuhan, China); the purity of the peptides was >80%.

### 4.12. ICS

ICS was performed on splenocytes isolated from immunized BALB/c mice. For the assay, 2 × 10^6^ cells were plated into the wells of 24-well culture plates (TPP, Trasadingen, Switzerland) and stimulated with the peptide mixture indicated above. Each peptide was added at a concentration of 20 μg/mL per well, and cells were incubated for 4 h at 37 °C in the presence of 5% CO_2_ and for an additional 16 h with Brefeldin A (5 μg/mL, GolgiPlug BD Biosciences). The next day, the cells were stained with anti-CD3 conjugated to Alexa Fluor 700 (BD), anti-CD4 conjugated to BV786 (BD), and anti-CD8 conjugated to FITC (BD); fixed with 1% paraformaldehyde in PBS; and permeabilized with 0.5% Tween 20 in PBS according to the manufacture’s instructions. Then, the cells were stained to detect intracellular cytokines anti-IFN-γ APC (BD, USA). The samples were analyzed by using a ZE5 flow cytometer (Bio-Rad) and the Everest software.

### 4.13. Software and Statistical Analysis

Data were analyzed by using GraphPad Prism 6.0 software. Differences between groups were determined by using the nonparametric Mann–Whitney method; *p* < 0.05 was considered statistically significant.

Images in Figure 2(A1,B1,G) and Figure 3A were created with biorender.com.

## Figures and Tables

**Table 1:** Peptides from the RBD protein for stimulation of splenocytes from BALB/c mice in ELISpot and ICS.

Number	Sequence	MHC Restriction
1	VYAWNRKRI	H2-Kd
2	FERDISTEI	H2-Ld
3	CGPKKSTNL	H2-Dd
4	KNIDGYFKIYSKHTP	H2-IEd
5	RFASVYAWNRKRISN	H2-IEd, H2-IAd
6	VGGNYNYLYRLFRKS	H2-IEd
7	GGNYNYLYRLFRKSN	H2-IEd
8	YNYKLPDDFTGCVIA	H2-IEd
9	NATRFASVYAWNRKR	H2-IEd, H2-IAd
10	KNKCVNFNFNGLTGT	H2-IEd
